# Coexistence of bullous pemphigoid, intrahepatic cholangiocarcinoma, and alopecia areata: a case report of multifactorial autoimmunity in a surgical context

**DOI:** 10.3389/fimmu.2025.1650253

**Published:** 2025-08-21

**Authors:** Shanlin Li, Ying Wang, Dingquan Yang

**Affiliations:** 1 Graduate School, Beijing University of Chinese Medicine, Beijing, China; 2 Department of Dermatology, The National Center for the Integration of Traditional Chinese and Western Medicine, China-Japan Friendship Hospital, Beijing, China

**Keywords:** bullous pemphigoid, intrahepatic cholangiocarcinoma (ICC), alopecia areata (AA), autoimmune, case report

## Abstract

**Background:**

Bullous Pemphigoid (BP) is caused by a predominantly Th2-mediated attack on the basement membrane by the production of anti-BP180 and anti-BP230 antibodies. Malignant tumors can exacerbate immune disorders through a variety of potential pathways, including pro-inflammatory responses in the tumor microenvironment, cross-immune responses induced by tumor-associated antigens, and the lifting of immunosuppressive states and activation of underlying autoimmune responses after surgery. Alopecia Areata (AA) is an autoimmune disease caused by T-lymphocyte-mediated destruction of the immune privilege of the hair follicle, specifically involving the immune axes of Th1, Th2 and Th17. Both AA and BP are associated with dysregulation of cytokines such as IL-4, IL-13, and IL-17. However, the mechanisms underlying the coexistence of the three are unclear, and no cases of their combination have been reported.

**Case presentation:**

A 67-year-old male patient presented to the clinic complaining of scattered erythema and blisters on the trunk and extremities with marked itching for 4 days. Previously, the patient had undergone surgery for intrahepatic cholangiocarcinoma 10 days earlier. Furthermore, he had developed alopecia areata with the SALT 50 six months earlier and has recovered with white hairs. By combining the patient’s history with his laboratory tests and pathologic examinations, the patient was diagnosed with bullous pemphigoid, intrahepatic cholangiocarcinoma, and alopecia areata. The patient demonstrated normalization of serum tumor markers post-resection of intrahepatic cholangiocarcinoma. Bullous pemphigoid lesions resolved completely with dupilumab-targeted therapy, while alopecia areata exhibited spontaneous remission with full hair regrowth despite no disease-specific treatment.

**Conclusion:**

This case report is the first to present the coexistence of bullous pemphigoid, malignant tumors, and alopecia areata, especially since the patient did not undergo immune medication, such as chemotherapy, which has implications for clinical confrontation with the combined presence of these diseases.

## 1 Introduction

Bullous pemphigoid (BP), an autoimmune subepidermal blistering disorder, manifests clinically as tense bullae with severe pruritus, often accompanied by urticarial or eczematous lesions (1). Its hallmark features include autoantibodies targeting BP180/BP230 antigens and complement C3 deposition at the dermoepidermal basement membrane. Pathological Th2, Th17, and Tfh cell expansion promotes IL-4/IL-17-mediated inflammation, while Treg dysfunction facilitates autoreactive CD4+ T cell activation and autoantibody production (2).

Malignancies induce multilevel immune dysregulation, with tumor microenvironments fostering systemic immunosuppression (3). Postoperative immune reconstitution following tumor resection may paradoxically trigger latent autoimmune responses. Recent systematic reviews have failed to establish a direct association between malignancy and bullous pemphigoid (4). However, compelling evidence has emerged linking BP development to immune checkpoint inhibitor (ICI) therapy in cancer patients (5). Notably, immune checkpoint inhibitors (ICIs) have been epidemiologically linked to BP development, with 0.3–1% of ICI-treated patients exhibiting this complication (6, 7). A documented case described BP onset following neoadjuvant chemotherapy and surgical resection in a patient with intrahepatic cholangiocarcinoma, which may suggest potential synergistic effects of cytotoxic therapy and surgical stress in triggering autoimmune phenomena (8).

Alopecia areata (AA) is an autoimmune disorder characterized by non-scarring hair loss, primarily driven by the collapse of hair follicle immune privilege. The pathogenesis involves follicular secretion of IL-2, IL-15, and IFN-γ, which activate cytotoxic CD8+ T cells. These cells, with CD4+ T cell assistance, infiltrate the perifollicular microenvironment, inducing Th1/Th2 immune imbalance and inflammatory cascades that culminate in hair loss. Th17 lymphocytes exacerbate AA-associated immune dysregulation through pro-inflammatory cytokines (e.g., IL-17, IL-22) and disruption of Treg cell homeostasis (9, 10).Emerging evidence suggests that the shared immunopathogenic mechanisms underlying alopecia areata predispose patients to concurrent autoimmune or immune-mediated dermatoses, including vitiligo, psoriasis, and atopic dermatitis (11–13). However, the co-occurrence of AA with BP remains exceptionally rare in clinical practice (14). Notably, previously reported cases of AA-BP comorbidity have been documented exclusively in the context of specific pharmacologic triggers, particularly immunomodulatory agents (15, 16).

To date, no studies have reported the coexistence of BP, malignancy and AA. For the first time, this case describes an intrahepatic cholangiocarcinoma patient with a six-month AA history who developed BP one week postoperatively without prior exposure to immunologic agents such as ICIs, providing novel insights into shared immunopathogenic mechanisms underlying this triad.

## 2 Case presentation

### 2.1 Clinical timeline

#### 2.1.1 AA

**Figure 1:**
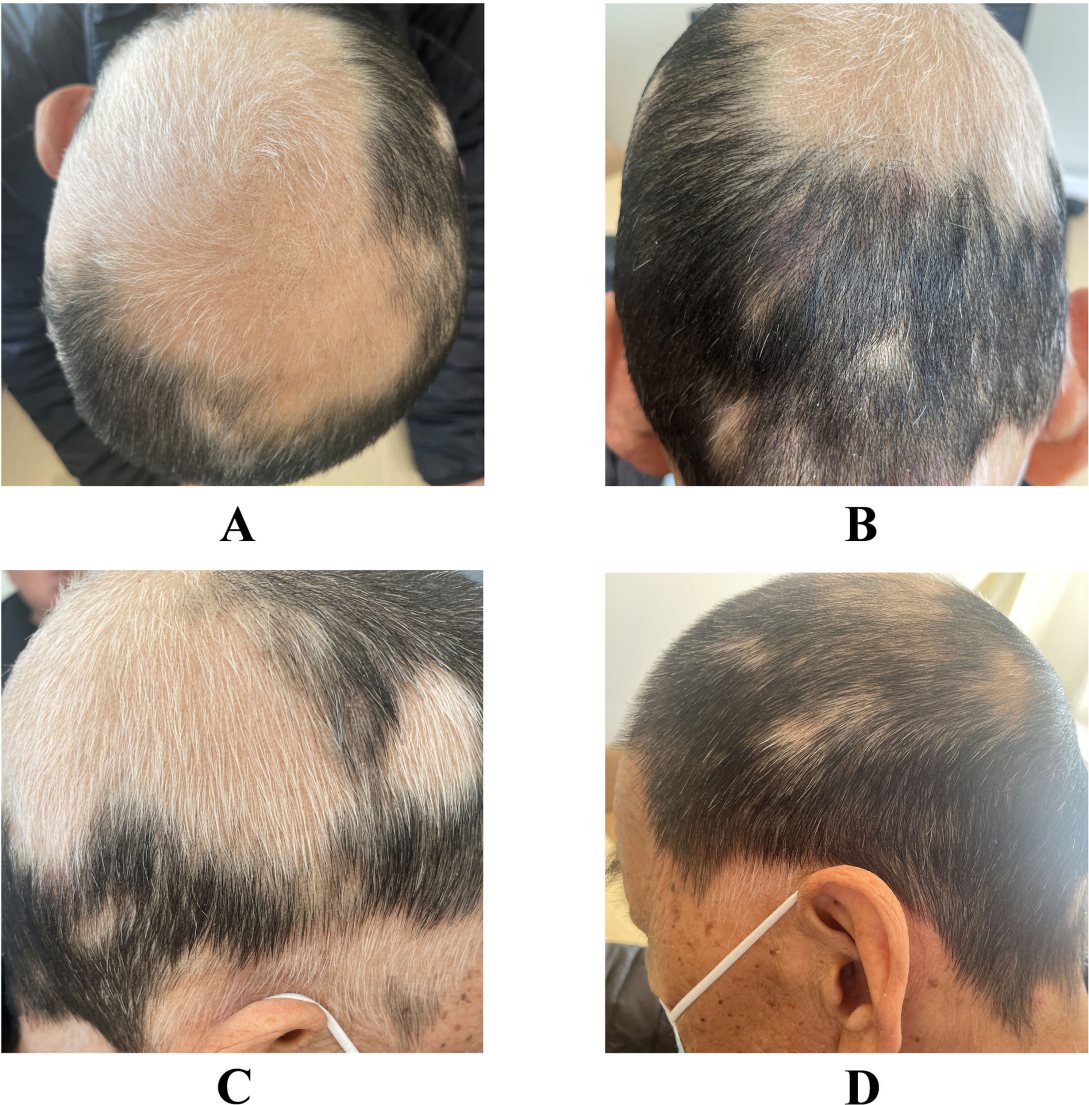
Alopecia areata manifestation of the patient with spontaneous regrowth of white hairs. **(A)** Top region. **(B)** Occipital region. **(C)** Right temporal region. **(D)** Left temporal region.

Six months ago, the patient had patchy hair loss without obvious triggers, accounting for about 50% of the total head area, which was not treated. Approximately two months following disease onset, hair regrowth was observed in the alopecic patches, demonstrating universal depigmented regrowth. At the current consultation, physical examination revealed a normo-pigmented scalp without erythema, scaling, or other cutaneous abnormalities. Large patches of white hair were prominently distributed, interspersed with smaller focal areas of leukotrichia across the entire scalp. Based on the characteristic clinical progression and examination findings, a definitive diagnosis of alopecia areata (AA) was established (**Figure 1**).

#### 2.1.2 Intrahepatic cholangiocarcinoma

**Figure 2:**
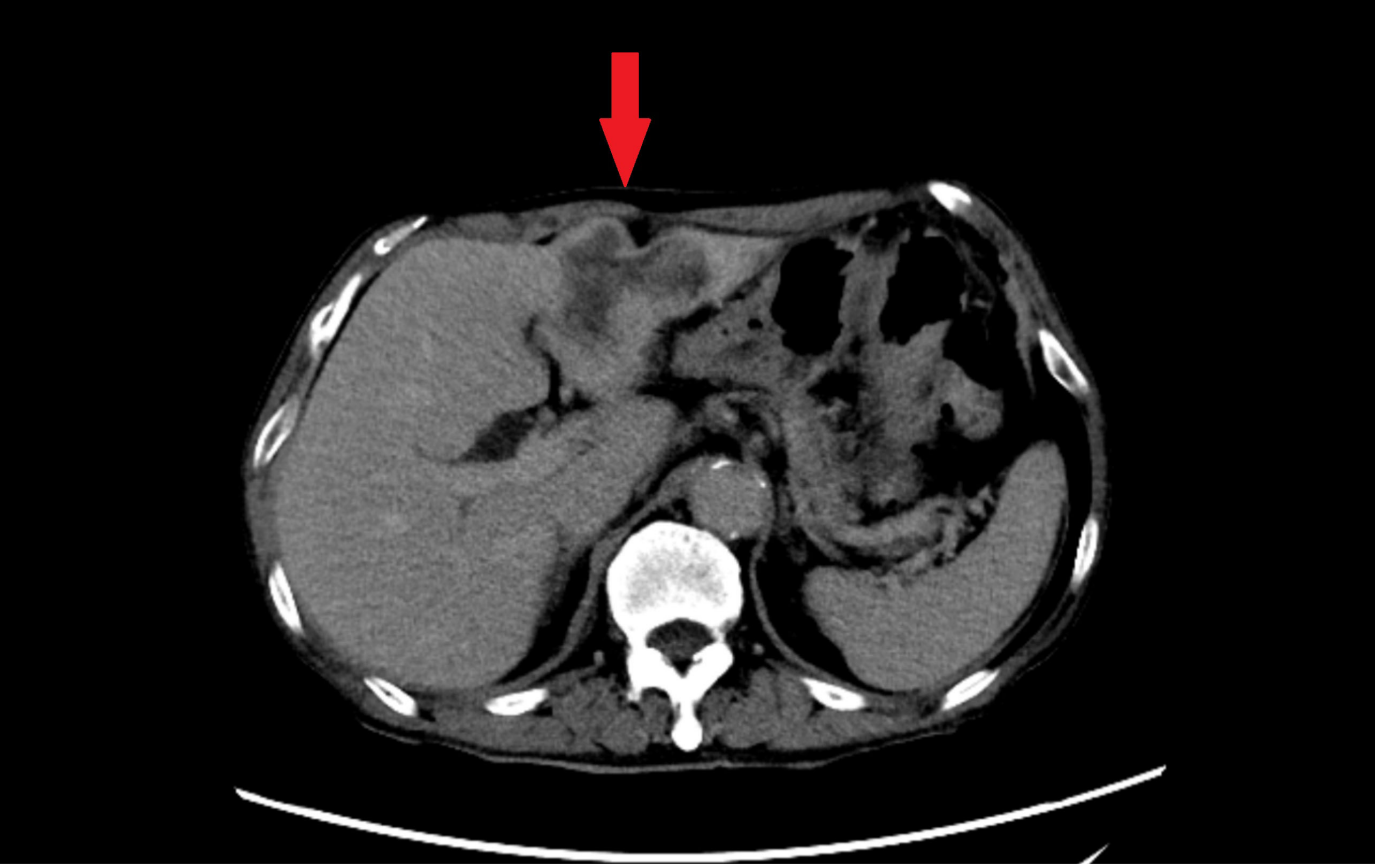
Abdominal contrast-enhanced CT in the equilibrium phase. Axial imaging demonstrates an irregular, heterogeneously enhancing mass in the left hepatic lobe (red arrow), accompanied by adjacent bile duct dilatation.

More than 1 month ago, the patient was admitted to the local hospital because of increasing abdominal pain lasting for 22 days, and ultrasound and abdominal CT revealed a space-occupying lesion in the left lobe of the liver (**Figure 2**). 17 days ago, a liver puncture was performed in our hospital, and the pathology showed adenocarcinoma. Meanwhile, serum tumor marker profiling revealed significantly elevated preoperative levels of CA125(59.3 U/mL), CA19-9(1355 U/mL), and CA15-3(36.1 U/mL). 8 days ago, laparoscopic left hemihepatectomy with abdominal lymph node dissection was performed under general anesthesia, and the pathology showed intrahepatic cholangiocarcinoma after the operation. The final oncological diagnosis of the patient was intrahepatic cholangiocarcinoma, with a stage of T2bN1M0, and it was classified as stage IIIB. Postoperative assessment demonstrated marked reduction in all three markers, with CA125(32.8 U/mL, reference range<35.0) and CA15-3(12.6 U/mL, reference range ≤ 25.0) normalizing to reference ranges. Other tumor markers remained within normal limits. Five weeks after the surgery, the patient began the first cycle of adjuvant chemotherapy. After chemotherapy, bone marrow suppression occurred. The blood routine test conducted at another hospital showed severe reduction in neutrophils and platelets. The patient was hospitalized at that hospital for platelet transfusion and the subsequent chemotherapy plan was suspended.

#### 2.1.3 Bullous pemphigoid

**Figure 3:**
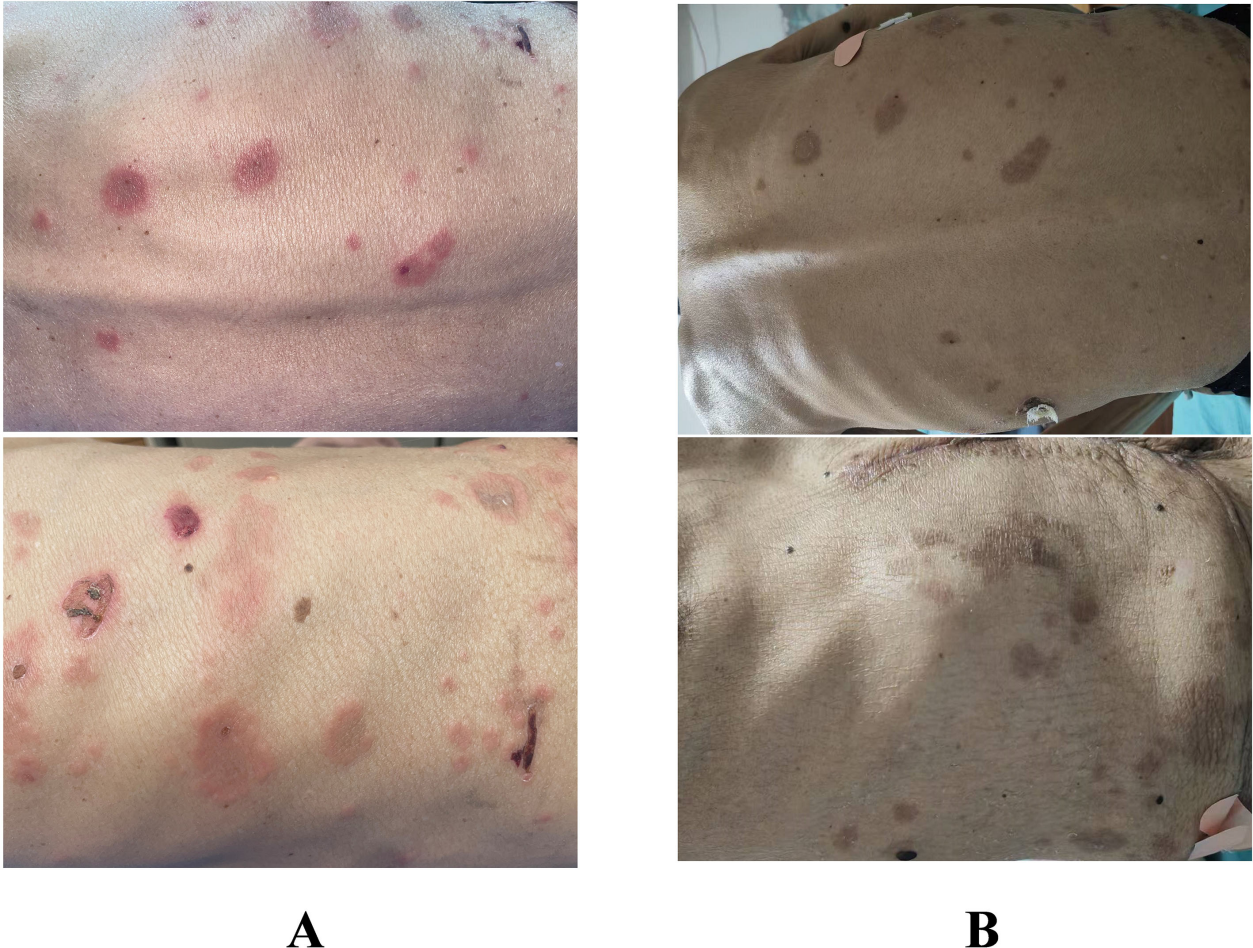
Presentation of the patient’s lesions. **(A)** Pre-treatment lesions. **(B)** Post-treatment lesions.

One week after surgery, the patient presented to our dermatology department with scattered skin lesions, before chemotherapy had been initiated and no other immunologic agents had been administered. The patient complained of scattered erythema and blisters on the trunk and extremities with itching for 3 days, and the lesions had been progressively increasing over the past 3 days. (**Figure 3**) The physical examination showed that Nikolsky’s sign was negative, and no mucosal involvement was observed throughout the body.

Laboratory tests showed that the patient’s total IgE was 55.3 IU/mL (within normal range of 5–161 IU/mL) and the total number of eosinophils was 0.28*10^9/L (within normal range). Autoantibody panel showed Dsg-1 and Dsg-3 are negative, anti-BP180 antibody was >500.00 AU/mL (markedly elevated), and anti-BP230 antibody was within normal limits.

**Figure 4:**
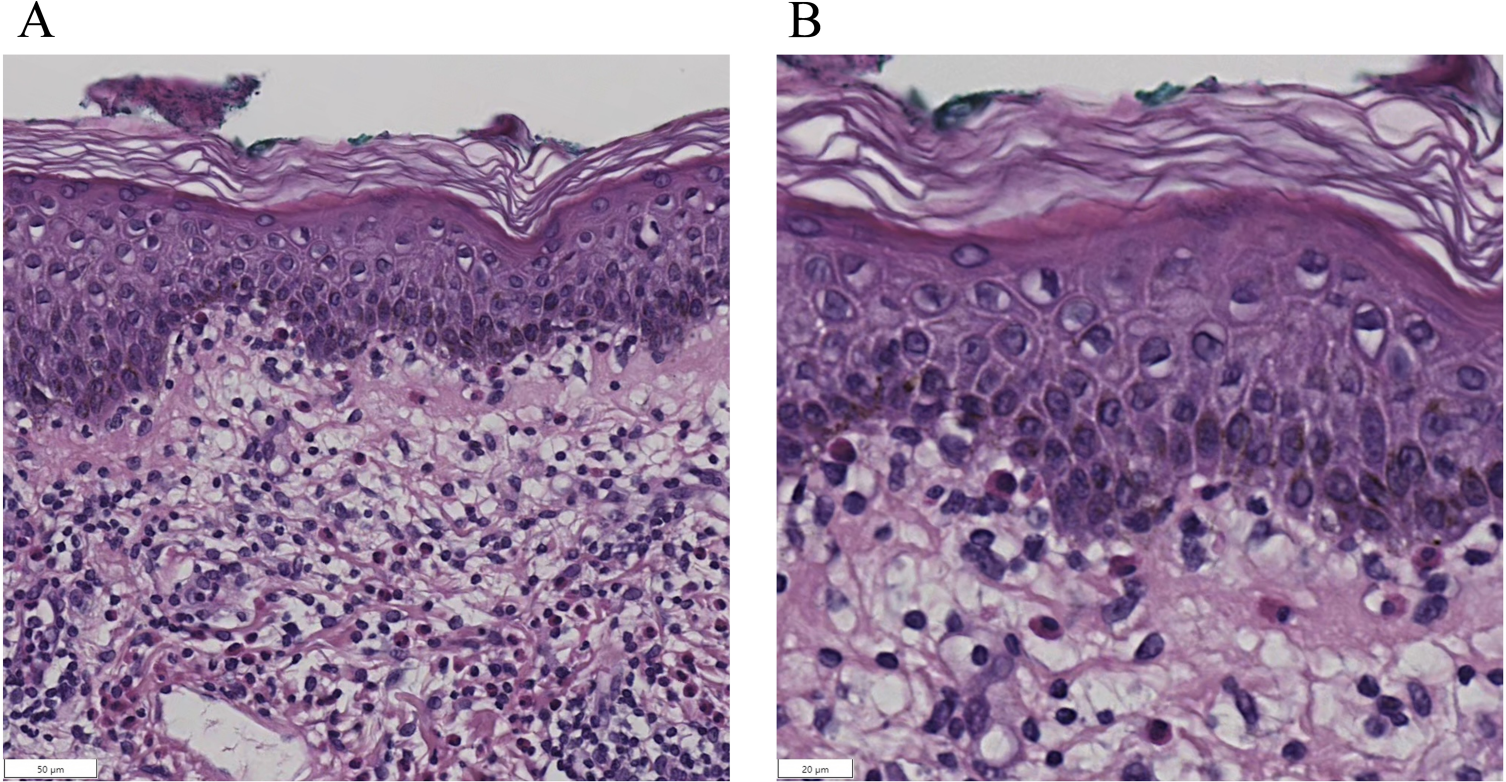
Histopathological examination of skin biopsy (hematoxylin-eosin staining). Subepidermal blister formation is observed, with lymphocytes and eosinophils within the blister cavity. The dermoepidermal junction exhibits edema and a mixed inflammatory infiltrate composed of lymphocytes and eosinophils. **(A)**. Low-magnification view (×200). **(B)**. High-magnification view (×400).

Pathological examination was performed on the edematous erythema area. Dermatopathologic biopsy showed the dermal-epidermal junction was edematous with lymphocytes, eosinophils and infiltration of lymphocytes and eosinophils. (**Figure 4**) Although no obvious subepidermal blisters were observed under the microscope, the above manifestations were consistent with those of the erythematous stage of BP. Combined with the above history, laboratory tests and pathologic findings, the diagnosis of bullous pemphigoid pemphigus was considered (17).

### 2.2 Therapy

The patient was initiated on subcutaneous dupilumab with an initial loading dose of 600 mg, followed by 300 mg administered every two weeks. A. Concurrent topical therapy included halobetasol cream (twice daily) applied to affected areas. Oral medications comprised minocycline hydrochloride capsules (100 mg twice daily). Dupilumab is a biological agent that can target the pathogenesis of BP. It can reduce the adverse reactions associated with cumulative exposure to corticosteroids and conventional immunosuppressants (1). Considering the patient’s tumor background and the fact that the patient’s BP was not particularly severe, oral corticosteroids were not given to the patient, and minocycline in combination with dupilumab was used instead (17).

After half a month of treatment, the patient’s rash improved significantly compared with before. (**Figure 3**) The erythema became darker, most of the blisters disappeared, the itching was significantly reduced, and there was no new rash.

At the time of the patient’s visit with BP, complete regrowth of hair had been achieved in the area of alopecia areata, which was before the initiation of dupilumab therapy for BP. Spontaneous hair regrowth occurred in all AA-affected regions without therapeutic intervention. However, all regenerated hairs were gray. And no emergence of new alopecic patches during follow-up. The patient was asked to follow the original program to continue treatment for one month and then return to the clinic.

## 3 Discussion

The coexistence of autoimmune dermatoses and malignancies presents a complex interplay of immune dysregulation, environmental triggers, and shared pathogenic mechanisms. This case report highlights the first documented instance of alopecia areata (AA) and bullous pemphigoid (BP) co-occurring in a patient with cholangiocarcinoma, independent of immunomodulatory therapies such as immune checkpoint inhibitors (ICIs). The underlying immunopathology likely involves overlapping Th1/Th17 and Th2-driven inflammatory pathways, granzyme B-mediated cytotoxicity, and postoperative immune reconstitution.

Bullous pemphigoid (BP), the most prevalent autoimmune bullous dermatosis (AIBD), is characterized by autoantibodies targeting BP180 and BP230 antigens, clinically presenting with tense bullae on the trunk/limbs, severe pruritus, and laboratory findings of eosinophilia. The association between malignancy and autoimmune diseases is well-established, with cancer patients exhibiting increased risks of paraneoplastic syndromes and immune-mediated comorbidities (18). Immune checkpoint inhibitors (ICIs), widely employed in treating solid malignancies, are recognized for inducing immune-related adverse events (irAEs), with cutaneous manifestations being the most prevalent following PD-1/CTLA-4 blockade. The pathogenesis of ICI-associated irAEs is primarily attributed to cytotoxic T-cell activation against self-antigens, triggering autoimmune reactions (19).

Notably, this patient was more susceptible to misdiagnosis as paraneoplastic pemphigus (PNP) than bullous pemphigoid (BP) due to their partial clinical overlap, as both conditions may present with vesicles, bullae, pruritus, and potential mucosal involvement (20). However, while PNP typically manifests with more prominent and severe mucosal lesions, this patient exhibited no mucosal involvement, making clinical differentiation challenging. The definitive distinction relies on serological and histopathological findings. Serologically, BP is characterized by autoantibodies against BP180 and BP230, whereas PNP demonstrates a broader autoantibody profile, often including elevated desmoglein antibodies, but in this case, the Dsg antibody was not elevated (21). Histopathologically, BP typically presents with subepidermal blistering and dermal eosinophilic infiltration, while PNP shows greater complexity, featuring subepidermal blisters combined with intraepidermal acantholysis, interface dermatitis, and keratinocyte necrosis (21, 22). The patient’s favorable response to dupilumab and minocycline combination therapy, along with an excellent prognosis, further supports the BP diagnosis, as PNP would likely have been more refractory with a poorer outcome. Although both BP and PNP occurring in cancer patients may represent paraneoplastic phenomena, PNP exhibits a well-defined and strong association with malignancy, whereas the relationship between BP and concurrent tumors remains weaker and more ambiguous, with neoplasms potentially acting as incidental or exacerbating factors rather than exhibiting the characteristic high prevalence seen in PNP.

A Japanese case report described an elderly male with intrahepatic cholangiocarcinoma who developed BP 24 days postoperatively after 9 months of neoadjuvant therapy (gemcitabine + cisplatin + durvalumab) (8). In stark contrast, our patient developed BP one week after surgery without prior exposure to ICIs or other immunomodulatory therapies. Our findings support a novel hypothesis of postoperative immune rebound, potentially involving tumor-derived immunomodulatory signals or surgical trauma-induced cytokine storms. A study has combed through the suppression of regulatory T cells (Tregs) function after transplant surgery (23). Based on this, it is reasonable to speculate whether cancer surgery also causes Treg cells dysfunction, which can lead to autoimmune skin diseases such as BP. Unfortunately, this patient did not undergo the examination of Treg cells and their related cytokines. Subsequent multi-sample testing can be conducted to further explore the pathogenesis.

A cross-sectional study by Xie D et al. investigating the epidemiology of alopecia in autoimmune bullous dermatoses (AIBD) revealed that over 70% of AIBD patients exhibited at least one subtype of hair loss, though no cases of concurrent BP and AA were documented (14). Notably, Someili A et al. reported a rare case of AA coexisting with BP triggered by DPP-4 inhibitor therapy (15). In contrast, our patient had no history of diabetes or exposure to such pharmacologic agents. Critically, our case highlights malignancy and surgical intervention as potential immunomodulatory triggers, with BP emerging one week postoperatively in the patient with preexisting AA and cholangiocarcinoma.

Alopecia areata (AA) arises from gene-environment interactions driving autoimmune-mediated patchy hair loss. The pathological nexus between AA and BP may reside in shared autoimmune mechanisms. Both disorders exhibit Th1/Th17-driven inflammation, marked by upregulated Th1/Th17 subsets, excessive IL-17/IL-23 secretion, and dysfunctional Treg cell regulation. Distinctively, BP demonstrates a Th2-polarized immune response against BP180/BP230, recruiting eosinophils to amplify inflammation (24). Intriguingly, emerging evidence implicates Th2 pathways in AA pathogenesis, with recent clinical trials evaluating Th2-targeted biologics (e.g., dupilumab) for AA management (25, 26), suggesting Th2 dysregulation as a potential immunobiological link between these entities. Furthermore, granzyme B, a serine protease with established causal roles in BP pathogenesis through epidermal detachment induction, has recently emerged as a diagnostic and prognostic biomarker in AA, highlighting its broader implications in autoimmune dermatoses (27). This shared granzyme B-mediated cytotoxicity may further bridge the immunopathological gap between BP and AA.

Regarding the therapeutic approach in this case, we employed a regimen combining oral minocycline with subcutaneous dupilumab, supplemented by topical corticosteroids and oral antihistamines. Minocycline, a tetracycline antibiotic, has been validated as a safe and effective agent for BP management. Dupilumab, a biologic targeting IL-4/IL-13 receptor blockade, selectively inhibits Th2-mediated immunity, exerting specific therapeutic effects on BP without systemic immunosuppression (1). This Th2-specific mechanism may explain the lack of improvement in AA, which is predominantly driven by Th1/Th17 pathways.

Complete yet depigmented hair regrowth may reflect melanocyte-targeting immunity in AA, wherein inflammatory cascades preferentially disrupt follicular melanogenesis despite hair cycle restoration. Notably, hair follicles in the anagen phase, characterized by active melanogenesis, demonstrate heightened susceptibility to autoimmune attack in alopecia areata (28, 29). During early hair regrowth, it has been proven that reduced melanoblast recruitment and aberrant melanin synthesis occur within regenerating follicles (30, 31). Recent comparative studies of AA and vitiligo further implicate melanocyte pathology from a stem cell biology perspective, suggesting shared disruptions in the follicular melanocyte unit and melanocyte stem cell niche maintenance despite distinct clinical phenotypes (32).

While prior reports have described cases of coexisting AA, BP, and malignancy, this represents the first instance not attributable to immunomodulatory drug exposure. This case underscores the necessity for heightened vigilance against autoimmune dermatoses in malignancy patients with preexisting autoimmune disorders, irrespective of immunotherapy use. However, there are inevitable shortcomings in this case report. Out of respect for the patient’s preference and to uphold patient-centered care principles, only a single tissue sample was obtained for histopathological examination without direct or indirect immunofluorescence testing, representing a diagnostic limitation in this case. Serial monitoring of BP antibody titers was not possible post-treatment due to the patient’s commitment to chemotherapy and their own assessment of significant clinical improvement rendering re-testing undesirable. More detailed longitudinal assessment of outcomes (including Treg analysis) and obtaining the patient’s perspective were limited by the patient’s personal circumstances and prioritization of oncological management following the significant improvement of skin disease. These limits our ability to correlate serologic responses with clinical responses and impede further exploration of the mechanisms underlying this case. Further mechanistic studies are warranted to elucidate the interplay between tumor biology, postoperative immune reconstitution, and autoimmune dermatologic cascades in this clinical trial.

## 4 Conclusion

This case presents the first documented coexistence of bullous pemphigoid (BP), intrahepatic cholangiocarcinoma and alopecia areata (AA) in a patient without prior immunomodulatory therapy, highlighting postoperative immune rebound as a potential trigger for autoimmune dermatoses. However, the specific immune mechanisms underlying the combination of these diseases are unknown.

Clinicians should maintain heightened vigilance for autoimmune dermatoses in cancer patients, particularly those with preexisting immune dysregulation. Future studies should delve into the immune mechanisms of the combination of the three diseases.

## Data Availability

The raw data supporting the conclusions of this article will be made available by the authors, without undue reservation.
