# Correction: Coexistence of bullous pemphigoid, intrahepatic cholangiocarcinoma, and alopecia areata: a case report of multifactorial autoimmunity in a surgical context

**DOI:** 10.3389/fimmu.2025.1765423

**Published:** 2025-12-17

**Authors:** Shanlin Li, Ying Wang, Dingquan Yang

**Affiliations:** 1 Graduate School, Beijing University of Chinese Medicine, Beijing, China; 2 Department of Dermatology, The National Center for the Integration of Traditional Chinese and Western Medicine, China-Japan Friendship Hospital, Beijing, China

**Keywords:** bullous pemphigoid, intrahepatic cholangiocarcinoma (ICC), alopecia areata (AA), autoimmune, case report

An incorrect **Funding** statement was provided as “National High Level Hospital Clinical Research Funding (No. 2023-NHLHCRF-YYPP-TS-06)”. The correct funder is “China-Japan Friendship Hospital”, the correct funding statement reads: “The author(s) declare financial support was received from China-Japan Friendship Hospital (2021-HX-42) for the research and/or publication of this article”.

The original version of this article has been updated.

